# Association between preoperative lactate level and early complications after surgery for isolated extremity fracture

**DOI:** 10.1186/s12891-024-07409-x

**Published:** 2024-04-23

**Authors:** Yusho Nishida, Ryo Yamamoto, Soichiro Ono, Junichi Sasaki

**Affiliations:** Department of Emergency and Critical Care Medicine, Keio University School of Medicine, 35 Shinanomachi, Shinjuku, Tokyo, 160-8582 Japan

**Keywords:** Lactate, Post-operative complication, Isolated extremity fracture, Surgery, Timing

## Abstract

**Background:**

The role of lactate level in selecting the timing of definitive surgery for isolated extremity fracture remains unclear. Therefore, we aimed to elucidate the use of preoperative lactate level for predicting early postoperative complications.

**Methods:**

This was a single-center retrospective observational study of patients with isolated extremity fracture who underwent orthopedic surgery. Patients who underwent lactate level assessment within 24 h prior to surgery were included. The incidence of early postoperative complications was compared between patients with a preoperative lactate level of ≥ 2 and < 2 mmol/L. Moreover, subgroup analyses were performed based on the time from hospital arrival to surgery and fracture type.

**Results:**

In total, 187 patients were included in the study. The incidence of postoperative complications was significantly higher in patients with a preoperative lactate level of ≥ 2 mmol/L than those with a preoperative lactate level of < 2 mmol/L. This result did not change after adjusting for age and severity. Further, a high preoperative lactate level was associated with a greater incidence of postoperative complications in patients who underwent definitive surgery within 6 h after arrival.

**Conclusion:**

A preoperative lactate level of ≥ 2 mmol/L was associated with a greater incidence of early postoperative complications in isolated extremity fractures. Nevertheless, this correlation was only observed among patients who underwent definitive fixation within 6 h after hospital arrival.

**Supplementary Information:**

The online version contains supplementary material available at 10.1186/s12891-024-07409-x.

## Introduction

Extremity fracture is a major public health issue, and surgical treatment and long-term rehabilitation are required to help patients continually perform activities of daily living [1]. Given the anticipated rise in extremity fractures owing to the aging population [2, 3], a multidisciplinary approach is essential to mitigate functional impairment post-injury [4]. Among various interventions, expedited bone fixation has been identified as one of the most efficacious treatments in various studies. as supported by multiple studies [5–8].

The mortality rate after hip fracture has reduced with bone fixation within 24 h after injury [9, 10]. However, data regarding the appropriate timing of open reduction and internal fixation (ORIF) for isolated extremity fracture are limited. Several management protocols for poly or severe trauma, such as early appropriate care, safe definitive surgery, and damage control orthopedics, have been extensively examined [9, 10]. Nevertheless, there is still no validated protocol for early ORIF that does not increase the incidence of postoperative complications among patients with an isolated extremity fracture. Furthermore, some studies investigated the application of preoperative hemodynamic status for developing a prognostic score for orthopedic surgery [11]. However, the application of these protocols for determining the appropriate timing of ORIF for isolated extremity fracture has not been confirmed.

Lactate is an excellent metabolic marker that is used indirectly to monitor resuscitation in poly-trauma [12]. Considering that serum lactate level has been used to determine the optimal timing of ORIF in patients with severe poly-trauma [13], elevated serum lactate levels could be useful for detecting insufficient resuscitation among patients with isolated extremity fracture. Therefore, it is convenient for decision-making regarding surgical timing. Hence, the current study aimed to elucidate whether lower preoperative lactate level than the certain threshold is a suitable indicator for the safety of orthopedic surgery. We hypothesized that confirming that preoperative lactate level is lower than the certain threshold before the initiation of definitive orthopedic surgery is associated with a lower incidence of early complications.

## Materials and methods

### Study design and setting

This was a single-center retrospective observational study conducted in 2021 at a university hospital in Tokyo, Japan. The institution is a tertiary care facility that handles all types of extremity injuries, including high-energy trauma and isolated bone fracture. The obtained data was collected from 2013 to 2020. The authors did not access to information that could identify individual participants after data collection. The data collection was performed from October 1st to 8th 2021. The data was accessed from October 12th to 29th 2021. This research has been approved by the Keio University School of Medicine, Ethics Committee (application number: 20,180,222). The requirement for informed consent was waived because of the anonymous nature of the data used.

At the study institution, lactate levels are measured on hospital arrival in all patients and serial measurements were conducted in some patients based on the decision of a treating physician. Patients with extremity fracture were either transported from the scene or transferred from other hospitals. These patients were initially assessed by emergency physicians and then assessed by an orthopedic surgeon within 30 min. During an initial assessment, trauma survey was done in accordance with ATLS (Advanced Trauma Life Support). Fluid resuscitation was conducted for patients with hemodynamic instability and blood products were transfused when continuous bleeding was observed. Hemodynamic monitoring continued until the ORIF. Management strategy for ORIF, including the necessity of external fixation and the timing of definitive surgery, was determined via a discussion among emergency physicians and orthopedic surgeons. Generally, the resources such as operating room (OR), basic orthopedic implants, and skilled surgeon were always available and most patients underwent definitive surgeries within 24 h after hospital arrival.

### Study population

Patients with extremity fracture who underwent orthopedic surgery were identified by reviewing medical records. The inclusion criteria were as follows: (1) patients aged ≥ 18 years; (2) those who underwent definitive bone fixation including ORIF, hemiarthroplasty, and arthroplasty; and (3) those who underwent lactate level assessment within 24 h before orthopedic surgery. Meanwhile, the exclusion criteria were as follows: (1) patients with poly-trauma, defined as an Abbreviated Injury Scale score of > 2, in the head, face, neck, chest, or abdomen; (2) those with severe injuries, defined as an Injury Severity Score (ISS) of > 16 [14]; (3) those who underwent external fixation before definitive bone fixation; and (4) those who had other surgical procedures prior to orthopedic surgery, including thoracotomy, laparotomy, and craniotomy.

### Data collection and definitions

The medical records of patients were reviewed and retrieved by both emergency physicians and orthopedic surgeons. The obtained data included demographics; comorbidities; vital signs upon hospital arrival; serum lactate level, type of blood sample (arterial vs. venous); time from hospital arrival to surgery; severity, mechanism, and site of injury; type of fracture (open or closed) and surgery; postoperative complications; length of hospital stay; and survival status at discharge.

Preoperative serum lactate level was defined as lactate value obtained within 24 h before orthopedic surgery. When they were measured multiple times, the most recent value was utilized. The threshold for lactate level was set as 2.0 mmol/L and high preoperative lactate was defined as lactate level of ≥ 2mmol/L based on previous studies for other diseases [15]. OR waiting time was defined as time between hospital arrival and the initiation of ORIF.

The postoperative complications included cerebral disease (cerebral infarction, cerebral hemorrhage, and other brain disorders), cardiac arrest, new-onset or exacerbated cardiovascular disease, hospital-acquired pneumonia, respiratory failure requiring oxygen therapy, acute kidney injury, urethral tract infection, acute liver injury, deep vein thrombosis/pulmonary embolism, gastric ulcer diagnosed via esophagogastroduodenoscopy, and mortality according to the Clavien-Dindo Classification (severer than grade 2) [16].

### Outcome measures

The primary outcome was the incidence of 30-day postoperative complications. The secondary outcomes were length of hospital stay and survival at discharge.

### Statistical analysis

Patients were divided into the high- or low-lactate groups based on a preoperative lactate level (high-lactate, ≥2mmol/L and low-lactate, <2mmol/L). The primary and secondary outcomes were compared between the two groups using the Chi-square test and the Mann–Whitney U test. Then, multivariate logistic regression analysis was performed to adjust for age, ISS, and fracture type, where the number of covariates were limited considering the small sample size.

Several sensitivity analyses were conducted. First, another multivariate logistic model was developed using backward stepwise methods that included all available covariates, such as sex, systolic blood pressure, heart rate, injury site, fracture type, Gustilo classification, type of surgery, and OR waiting time in addition to the covariates in the primary model. Second, venous lactate levels were adjusted to arterial ones by subtracting 0.2 mmol/L and the primary logistic model was examined [17, 18]. Third, patients with considerably minor injury such as phalange fracture (open/closed digit/toe fractures) were excluded and the primary logistic model was repeated. Additionally, post-operative complication rates were compared between those achieving and not achieving lactate clearance, wherein lactate clearance was defined as a decrease in lactate level to < 2.0 mmol/L from ≥ 2.0mmol/L .

Subgroup analyses were performed in the all patients who were divided based on OR waiting time (< 6 and ≥ 6 h) and fracture type (open vs. closed). In each subgroup analysis, complication rates were compared between patients with high and low lactate level after adjusting age, ISS, fracture type, Gustilo classification, and OR waiting time.

Descriptive statistics were presented as median (interquartile range [IQR]) or number [percentage]). Results were expressed as 95% confidence interval (CI). In the hypothesis testing, a two-sided α threshold of 0.05 was considered statistically significant. All statistical analyses were conducted using the Statistical Package for the Social Sciences software version 26 (IBM Corp., Armonk, NY).

## Results

**Fig. 1:**
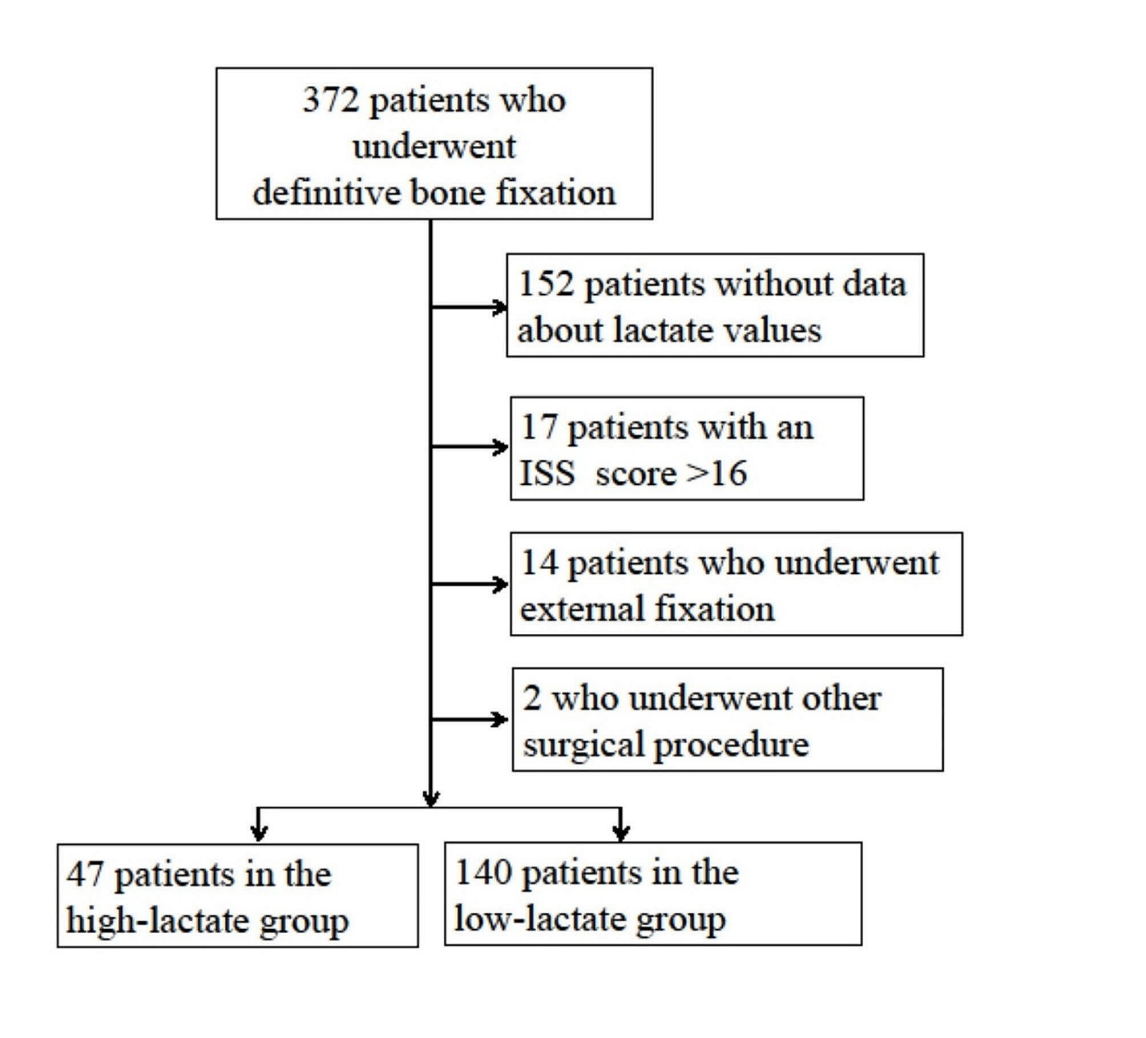
Patient flow diagram Of 372 patients who underwent definitive bone fixation, 187 were eligible for this study. Then, 47 and 140 were allocated to the high- and low-lactate group, respectively

A total of 187 patients with isolated extremity fracture underwent definitive bone fixation without external fixation and available preoperative lactate was eligible for this study (Fig. 1). Among them, 47 (25.1%) had high preoperative lactate. The patient characteristics were shown in Table 1. The lactate levels were 2.3 [2.1–2.8] mmol/L and 1.1 [0.9–1.5] mmol/L in the high- and low-lactate groups, respectively. The mean age and OR waiting time of the high- and low- lactate groups were 57 (44–74) and 78 (55–86) years; and 4.7 (3.2–9.9) and 5.2 (3.5–8.2) h, respectively. The numbers of open fractures were 25 (53.2%) in the high-lactate group and 47 (33.6%) in the low-lactate group. Details of postoperative complications were shown in Table 2.

Patients with high-lactate levels had a significantly greater incidence of postoperative complications than those with low-lactate levels (10 [21.3%] vs. 13 [9.3%], odds ratio [OR] = 2.64 [95% CI = 1.07–6.50], *p* = 0.04, Table 3), which remained in the multivariate logistic regression analysis (adjusted OR = 3.65 [1.37–9.74], *p* = 0.01, Table 3). Moreover, all sensitivity analysis, including another logistic regression model using all available covariates, revealed similar results (Table S1, S2 and S3). Lengths of hospital stay were comparable between the high- and low-lactate groups (16 vs. 17 days, Table 3) and no mortalities were observed in both groups.

In the subgroup analysis, a high preoperative lactate level was associated with higher incidence of 30-day postoperative complications in patients with an OR waiting time of < 6 h (OR = 4.87 [1.24–19.20], Table 4).

**Table 1:** Characteristics of patients with isolated extremity fracture

		High-lactate group		Low-lactate group		***p*** value
Case		47		140		
Age, years, median (IQR)		57	(44–74)	78	(55–86)	0.58
Sex, male, n (%)		29	61.7%	56	40.0%	0.01
Comorbidities, yes, n (%)		34	73.9%	116	83.5%	0.19
Mechanism, n (%)						
	Fall	28	62.0%	95	70.4%	0.37
	Traffic collision	13	29.0%	27	20.0%	0.30
	Penetrating	0	0.0%	4	3.0%	0.36
Vital signs upon arrival, median (IQR)						
	GCS	15	(15–15)	15	(15–15)	0.43
	RR, cycles/min	18	[16–20]	18	[16–20]	0.21
	HR, beats/min	89	(78–101)	79	(68–90)	0.01
	sBP, mmHg	140	(120–155)	142	(126–161)	0.56
	SpO_2_, %	98	(96–99)	97	(96–99)	0.33
Injury site, n (%)						
	Upper extremity	8	17.0%	18	12.9%	0.94
	Humerus	2	4.3%	1	0.1%	
	Ulnar/radius	4	8.5%	12	8.6%	
	Phalange	1	2.1%	0	0%	
	Other	1	2.1%	5	3.6%	
	Lower extremity	32	68.1%	144	102.9%	0.48
	Femur	21	44.7%	95	67.9%	
	Tibia/fibula	8	16.7%	28	20.0%	
	Phalange	0	0%	9	6.4%	
	Other	3	6.9%	12	8.5%	
Fracture type, open, n (%)		25	53.2%	47	33.6%	0.04
Crush injury		0	0.0%	0	0.0%	N/A
Injury severity, median (IQR)						
	ISS	9	(9–9)	9	(9–9)	0.81
	Ps	99	(97–99)	97	(97–97)	0.66
Lactate level, mmol/L, median (IQR)		2.3	(2.1–2.8)	1.1	(0.9–1.5)	0.23
Lactate in venous sample, n (%)		31	66%	58	41%	0.73
OR waiting time, h, median (IQR)		4.7	(3.2–9.9)	5.2	(3.5–8.2)	0.20
Type of surgery, n (%)						
	ORIF	39	84.8%	111	79.3%	0.68
	Hemiarthroplasty	5	10.9%	28	20.0%	0.19
	Arthroplasty	2	4.3%	0	0.0%	0.61

IQR = interquartile range, GCS = Glasgow coma scale, RR = respiration rate, HR = heart rate, sBP = systolic blood pressure, NA = not applicable, ISS = Injury Severity Score, Ps = probability of survival, ORIF = open reduction and internal fixation

**Table 2:** Details of major complications with isolated extremity fracture

		High-lactate group		Low-lactate group	
Cerebral disease, n (%)		2	4.5%	2	1.4%
Cardio-pulmonary, n (%)					
	Cardiac arrest	0	0.0%	1	0.7%
	Heart failure	0	0.0%	1	0.7%
	Arterial fibrillation	0	0.0%	1	0.7%
	Fat embolism	0	0.0%	1	0.7%
	Myocardial infarction	0	0.0%	1	0.7%
Gastrointestinal bleeding, n (%)		0	0.0%	1	0.7%
Liver injury, n (%)		5	10.6%	0	0.0%
Acute kidney injury, n (%)		1	2.1%	2	1.4%
Nosocomial infection, n (%)		3	6.4%	4	2.8%

**Table 3:** Postoperative complications and secondary outcomes

		High-lactate group	Low-lactate group	Odds ratio	95% CI	***p*** value
Postoperative complication, n (%)						
	Unadjusted	10/47 (21.3%)	13/140 (9.3%)	2.64	1.07–6.50	0.04
	Adjusted*			3.65	1.37–9.74	0.01
Length of hospital stay, days (IQR)		15.8 [7–21]	17.1 [8–23]			0.78
Survival at discharge, death, n (%)		0 (0.0%)	0 (0.0%)			N/A

CI = confidence interval, N/A = not applicable, IQR = interquartile range. *Multivariate logistic regression analysis of age, Injury Severity Score, and fracture type (open vs. closed) was conducted

**Table 4:** Postoperative complications in subgroup analysis

		Odds ratio*	95% CI
OR waiting time			
	< 6 h	4.89	1.22-19.67
	≥ 6 h	3.0	0.65-13.77
Type of fracture			
	Open fracture	0.62	0.05-7.31
	Closed fracture	5.68	1.88-17.13

CI = confidence interval, OR = operating room. *Multivariate logistic regression analysis to adjust for age, Injury Severity Score, fracture type (open vs. closed), Gustilo classification, and OR waiting time was conducted, in which odds ratio was calculated with low preoperative lactate as a reference

## Discussion

In this study, patients with a preoperative lactate level of ≥ 2 mmol/L had a significantly higher incidence of postoperative complications after definitive bone fixation for isolated extremity fracture than those with a preoperative lactate level of < 2 mmol/L. Notably, multivariate analyses after adjusting for age and severity of injury revealed the same results, that were also validated with several sensitivity analyses. Considering that short-term postoperative complications in isolated extremity fractures affected long-term functional outcomes [22, 23], the current results would propose further studies to examine the association between high lactate level and long-term functional consequences after extremity fractures.

Some pathophysiological mechanisms could be considered behind the association between higher lactate levels and a greater number of complications after orthopedic surgery in patients with isolated fracture. First, considering that the lactate level is elevated if hypoperfusion inhibits tissue oxygenation [19], a high-lactate level could indicate hidden blood loss even in patients with isolated extremity fracture [20]. Therefore, additional surgical insult was evident among patients with preoperative physiological instability alone [21]. Second, the threshold for high- and low-lactate level was set as 2.0 mmol/L, which is lower than that in studies of patients with poly-trauma (2.5–4.0 mmol/L) [9, 10]. A significant association between high-lactate levels and a greater number of postoperative complications could be attributed to the use of a lower threshold for differentiating patients with a high risk for bone fixation. Third, frailty, which is a background characteristic of patients, could reflect a high-lactate level [21]. This study included patients with fragility fractures caused by metabolic bone diseases, and serum lactate level could be a surrogate marker of pathophysiological vulnerability.

The association between high lactate levels and a greater number of postoperative complications was observed among patients who were transferred to the OR within 6 h. This result would be clinically useful to detect the premature surgery in isolated extremity fracture. In addition, patients with closed fracture showed the significant correlation between high lactate level and postoperative complications. Although the interpretation should be cautioned due to the small sample size, this result would suggest that relatively less-severe injuries may introduce post-operative complications if preoperative condition is disturbed.

Contrary, a higher preoperative lactate level did not have a significant influence on the length of hospital stay and survival status at discharge in this study. This could be caused by the inclusion of a small sample size and relatively less severe injury in the study population. The median length of hospital stay was ∼2 weeks, and no mortalities were identified. Moreover, as patients were only followed-up within a short period (maximum: 59 days) until hospital discharge, longer-term follow-up can identify any difference in mortality.

The current study had several limitations. First, the study setting could relatively be uncommon, where serum lactate level was measured in all isolated extremity fracture patients and most isolated fractures underwent definitive bone fixation within 24 h after arrival. Second, the preoperative lactate level obtained within 24 h prior to the surgery was used. Therefore, more frequent serial blood sampling could have different results [24]. Third, the current study did not examine other clinical parameters, such as base deficit [25], coagulopathy, and requirement of blood transfusion, which were considered valuable in decision-making regarding the timing of ORIF among patients with poly-trauma [9, 10]. Fourth, only a threshold value of 2.0 mmol/L was used based on previous studies for patients without fractures, and whether different thresholds can be applied remains unknown [26, 27]. Finally, this was a single-center retrospective study with a small sample size. Therefore, the results were not conclusive. Nevertheless, further prospective studies with a larger population should be performed to validate our results.

## Conclusion

A preoperative lactate level of ≥ 2.0 mmol/L was associated with early postoperative complications in patients with isolated extremity fracture. As such association was observed in patients with an OR waiting time of < 6 h, a lactate < 2.0 mmol/L would be recommended to be confirmed before ORIF for isolated extremity fracture.

### Electronic supplementary material

Below is the link to the electronic supplementary material.

Supplementary Material 1

Supplementary Material 2

Supplementary Material 3

## Data Availability

The datasets generated and/or analyzed during the current study are not publicly available because it was not permitted by study participants during consent process, but are available from the corresponding author on reasonable request under appropriate regulations.
