# Supplementary material for: Association between preoperative lactate level and early complications after surgery for isolated extremity fracture
Source: BMC Musculoskelet Disord. 2024 Apr 23;25:314. doi: 10.1186/s12891-024-07409-x (PMC11036590; doi:10.1186/s12891-024-07409-x)
Supplement: Supplementary file 1 — Supplementary Material 1 [file 12891_2024_7409_MOESM1_ESM.docx]

| Table S2. Postoperative complications in sensitivity analysis with adjusted lactate value and adjusted severity | | | | | |  |
| --- | --- | --- | --- | --- | --- | --- |
|  |  | High-lactate group | Low-lactate group | Odds ratio | 95% CI | p value |
| Postoperative complication, n (%) | |  |  |  |  |  |
|  | Adjusted lactate into arterial value* | 8/36 (22.0%) | 15/151 (9.9%) | 2.59 | 1.00–6.70 | 0.04 |
|  | Excluding patients only with minor injury** | 10/45(22.0%) | 13/132(9.8%) | 2.61 | 1.06-6.48 | 0.03 |
| CI = confidence interval. *Sensitivity analyses were performed with adjusted venous lactate value by subtracting 0.2 mmol/L **Sensitivity analyses were performed with adjusted severity of fracture by excluding isolated phalange fracture. | | | | | | |
|  |  |  |  |  |  |  |
