# Supplementary material for: Association between preoperative lactate level and early complications after surgery for isolated extremity fracture
Source: BMC Musculoskelet Disord. 2024 Apr 23;25:314. doi: 10.1186/s12891-024-07409-x (PMC11036590; doi:10.1186/s12891-024-07409-x)
Supplement: Supplementary file 2 — Supplementary Material 2 [file 12891_2024_7409_MOESM2_ESM.docx]

| Table S1. Multivariate logistic regression model* in sensitivity analysis | | | |  |
| --- | --- | --- | --- | --- |
|  |  | Odds ratio | 95% CI | p value |
| High preoperative lactate　≥　2.0mmol/L | | 3.36 | 1.29-8.74 | 0.01 |
| Age | | 1.01 | 0.99-1.04 | 0.34 |
| Sex (male sex) | | 2 | 0.72-5.52 | 0.18 |
| HR (tachycardia) | | 1.03 | 0.27-3.94 | 0.96 |
| sBP | | 0.99 | 0.97-1.01 | 0.24 |
| ISS | | 0.97 | 0.76-1.23 | 0.79 |
| Type of fracture (open vs. closed) | | 0.25 | 0.08-0.79 | 0.02 |
| Type of surgery | | - | - | - |
| Injury site | | - | - | - |
| Gustilo classification | | - | - | - |
| OR waiting time | | 1 | 1.00-1.00 | 0.25 |
| CI = confidence interval, HR = heart rate, sBP = systolic blood pressure, ISS = injury severity score, OR = operating room. *Multivariate logistic regression analysis with backward stepwise method was conducted, including age, sex, HR, sBP, ISS, type of fracture (open vs. closed), type of surgery, injury site, Gustilo classification ,and OR waiting time. Type of surgery, Injury Site, and Gustilo classification were not selected in backward stepwise method. | | | | |
|  |  |  |  |  |
