# Supplementary material for: Association between preoperative lactate level and early complications after surgery for isolated extremity fracture
Source: BMC Musculoskelet Disord. 2024 Apr 23;25:314. doi: 10.1186/s12891-024-07409-x (PMC11036590; doi:10.1186/s12891-024-07409-x)
Supplement: Supplementary file 3 — Supplementary Material 3 [file 12891_2024_7409_MOESM3_ESM.docx]

| Table S3. Postoperative complications in patients with and without lactate clearance* | | | | | |  |
| --- | --- | --- | --- | --- | --- | --- |
|  |  | Lactate clearance (+) | Lactate clearance (-) | Odds ratio | 95% CI | p-value |
| Postoperative complication, n (%) | | 2/12 (16.6%) | 10/47 (21.3%) | 0.74 | 0.14–3.94 | 0.72 |
| CI = confidence interval. *Lactate clearance was defined as a decrease in lactate level to < 2.0 mmol/L from ≥ 2.0mmol/L. | | | | | | |
